# Contagious Diseases Transferred to Mankind

**Published:** 1896-08

**Authors:** 


CONTAGIOUS DISEASES TRANSFERRED TO MANKIND. According to the statistics of the Imperial Hygienic Bureau for 1894, it is reported that in Germany aphthous fever was transmitted to mankind in many cases by using unboiled milk obtained from cows affected with aphthous fever; also anthrax in 109 cases. In most instances the infection occurred by slaughtering or skinning the animals; glanders in 3 cases, which ended fatally. —*Tierœrztliches Centralblatt.*

